# Determination of Material Strengths by Hydraulic Bulge Test

**DOI:** 10.3390/ma10010023

**Published:** 2016-12-30

**Authors:** Hankui Wang, Tong Xu, Binan Shou

**Affiliations:** China Special Equipment Inspection & Research Institute, Beijing 100084, China; dearwhk@foxmail.com (H.W.); xutong@csei.org.cn (T.X.)

**Keywords:** hydraulic bulge test, mechanical properties, small specimen

## Abstract

The hydraulic bulge test (HBT) method is proposed to determine material tensile strengths. The basic idea of HBT is similar to the small punch test (SPT), but inspired by the manufacturing process of rupture discs—high-pressure hydraulic oil is used instead of punch to cause specimen deformation. Compared with SPT method, the HBT method can avoid some of influence factors, such as punch dimension, punch material, and the friction between punch and specimen. A calculation procedure that is entirely based on theoretical derivation is proposed for estimate yield strength and ultimate tensile strength. Both conventional tensile tests and hydraulic bulge tests were carried out for several ferrous alloys, and the results showed that hydraulic bulge test results are reliable and accurate.

## 1. Introduction

Small specimen testing is a technology used to get the mechanical properties from a small volume material. Small punch test (SPT) is one of the most famous small specimen testing techniques and was first developed by nuclear industry in the 1970s to estimate the residual life of critical components [1,2]. After more than 40 years’ development, there are some standards in small punch tests, such as ASTM-F2183, GB/T-29459, and CEN CWA-15627 [3,4,5].

Until now, there still remain problems for the SPT, such as the friction factor between punch and small specimen [6]. Friction is a common phenomenon in the physical world, the friction factor is an experience parameter used to describe the interaction between the two contact bodies, and the friction factor is affected by type of materials, surface roughness and lubrication. It is noticed that friction plays a key role in small punch test results [7]. Another important issue is the method to obtain the material properties. Nowadays, most of the estimation methods for small punch test are based on empirical equations, which depend heavily on testing equipment, experience of scholars and a large amount experimental data, etc. [8]. Since the 1990s, with the development of computer simulation techniques, the inverse finite element method is used to calculate material properties [9,10]—that is, to use optimization methods to find material parameters that have the best similarity between the experimental data and the simulation results. The inverse finite element method is an improvement but it is also time consuming and not straightforward. 

The testing procedure and the material properties estimation method are the most important parts of the small specimen test technique. Compared with conventional tensile test, testing procedures and the estimation methods of material properties for hydraulic bulge test (HBT) and SPT are complicated, and restricted by the dimension of the specimen. For conventional tensile tests, the specimen size is restricted only by the material and the testing machine’s capacity. The geometry of the specimen is designed for a uniaxial tensile test, whereas the testing machine is designed to clamp and stretch the specimen. The situation is different for HBT and SPT, because the testing procedure is designed to satisfy a certain volume of the sample; therefore, HBT and SPT are much more complicated than conventional tests. In addition to the complexity of the SPT mechanical model, especially the friction model between punch and specimen, there is still no clear equation with solid theoretical background for the estimation of mechanical properties.

In this paper, the hydraulic bulge test method is introduced, which uses hydraulic oil make specimen deformation. First, there is no punch-specimen friction problem, as the specimen is in direct contact with hydraulic oil, which is a fluid, not a solid punch. Second, there is no punch influence to be concerned about, such as punch geometry or punch material. Third, mechanical deformation could be described by a mathematical model, so the tensile strength estimation are built on solid mechanical equations, whereas only specimen thickness and clamper size are considered in the equation. The drawback of the hydraulic method is testing environment—the test temperature range is limited by solidification and vaporization temperatures of hydraulic oil. Too high temperature will cause loading failure, whereas too low testing temperature will cause the seal part failure, and the leaking vapor may cause danger.

Both experimental method and estimation procedure will be introduced in this paper. The test equipment and the calculation method of the tensile properties are introduced in Section 2 and Section 3. The HBT results are shown in Section 4, which are verified with the data from conventional tensile tests. Section 5 is the conclusion of the paper.

## 2. Device and Experiment

**Figure 1:**
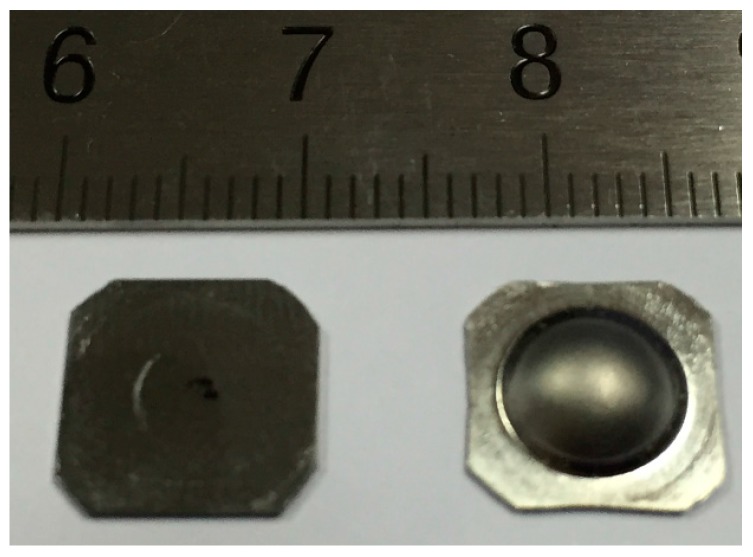
Specimen for hydraulic bulge test.

The specimen geometry is similar to that of small punch test, a small plain disk. The characteristic of HBT is hydraulic loading by high pressure oil. The shape of specimen for the hydraulic bulge test could be circular or square, depending on the sample size and the testing machine, with 10 mm in width and 0.5 mm in thickness. The square specimen is preferable because it has more area to be clamped, which means it is relatively easier to seal without oil leakage. The specimens used for hydraulic bulge test are shown in Figure 1. The left one is the specimen before test, and the right one is the specimen under 180 MPa hydraulic pressure.

**Figure 2:**
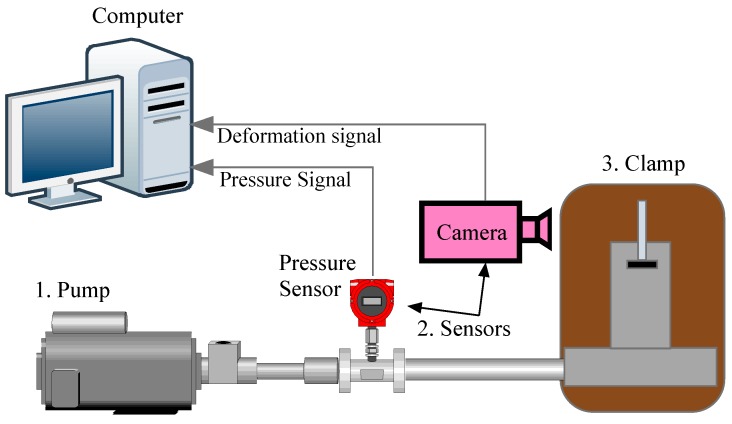
The hydraulic bulge test device.

The device used for HBT is complex in comparison to SPT, which consists of three key parts: a high-pressure pump, pressure and displacement sensors and specimen fixtures. The high-pressure pump, which acts as a driving force, is needed to reach at least 250 MPa or more for steel specimens. A pressure sensor and a displacement sensor are used in the system to pick up the pressure and the deformation signal, and the pressure-displacement curve is used to estimate material properties. The pressure sensor is widely used in industry, and a high accuracy is preferable. Since hydraulic oil with high pressure, at almost 200 MPa, suddenly jets out when there is a small specimen rupture, which will damage traditional contact displacement sensor, a non-contact displacement sensor, such as video gauge or laser extensometer, shall be used in the HBT system. All pressure signals and displacement signals are synchronized and recorded. The whole testing system is illustrated in Figure 2, in which the camera is used as video gauge to pick up the displacement signal.

**Figure 3:**
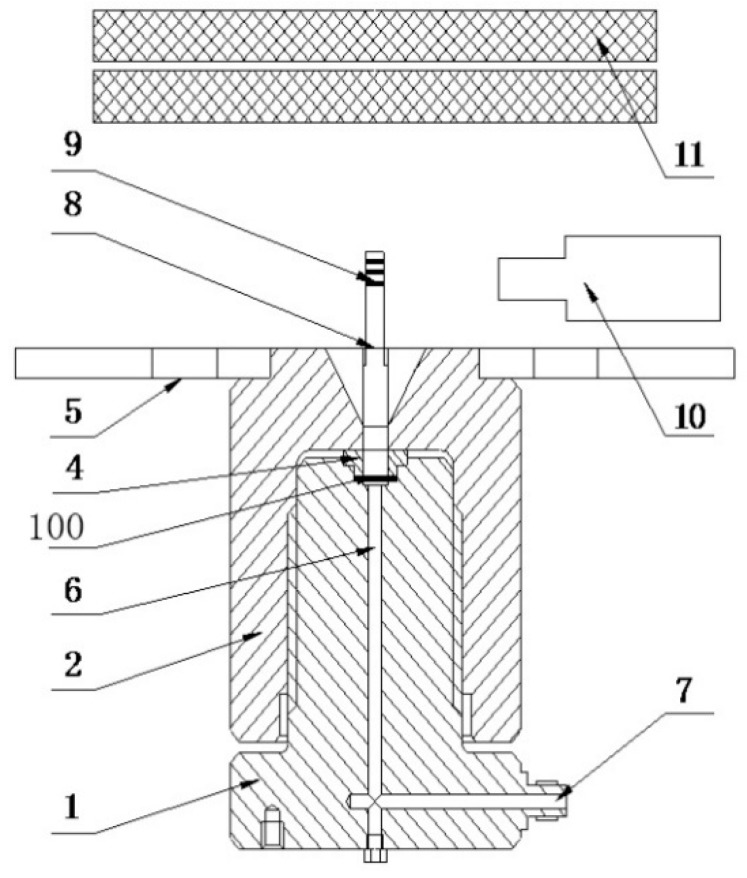
Fixtures: 1 base, 2 cap, 4 upper fixture, 5 handle, 6 oil channel, 7 oil inlet, 8 steel bar, 9 steel bar marker, 10 camera, 11 impact absorber, 100 O-ring seal.

A schematic diagram of the fixtures is shown in Figure 3. When the specimen deforms, the top of the specimen will push the steel bar (the eighth part in Figure 3) up forward, and then the camera captures the movement of the bar, and sends the video signal to a computer. The video gauge software analyzes the video signal to get the displacement of the steel bar and synchronizes with pressure signal to get the pressure-displacement curve. The diameter of the upper fixture hole and chamfering radius of the upper fixture (the fourth part in Figure 3) affect the pressure-displacement curve, and these two parameters are used for estimating the material properties. There is an impact absorber above the steel bar, with part number 11. When the specimen bursts, high pressure oil erupts, and the absorber is used for the erupted oil and steel bar. 

**Figure 4:**
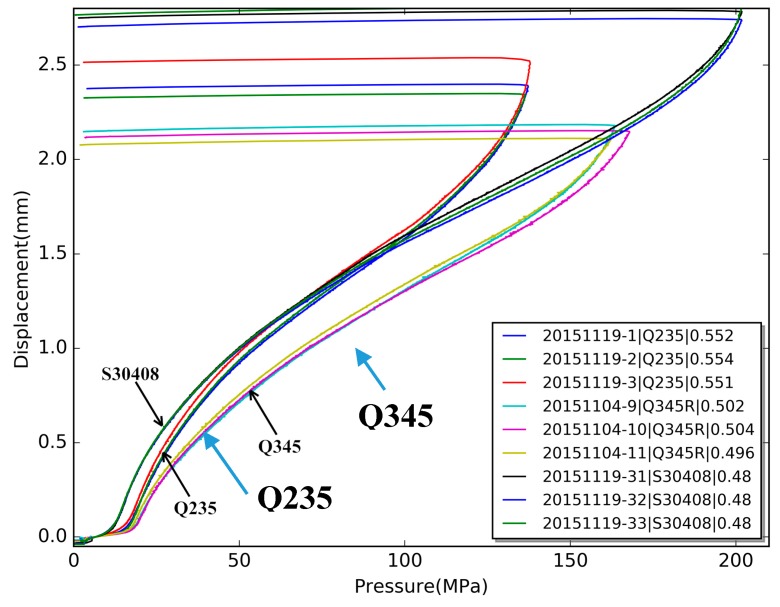
Pressure-displacement curves of hydraulic bulge test (HBT).

Figure 4 shows nine typical pressure-displacement curves for three grades of materials with thickness around 0.5 mm. The thickness of the S30408 specimen is 0.48 mm, and the thickness of the Q235 specimen is 0.55 mm. The curves in Figure 4 are divided into three groups. Obviously, no matter what kind of materials or how thick specimens are, pressure-displacement curves show the same pattern. At the beginning of loading—i.e., the pressure is less than a critical point—the curve increases gently. As the pressure increases beyond the critical point—named as the limitation pressure—the displacement increases rapidly. The limitation pressure of the specimen is different for different material. At the end of the loading, the displacement increases more and more quickly and eventually the specimen will rupture.

## 3. Theoretical Derivation

Two equations are proposed for estimating yield strength and ultimate tensile strength respectively. The equation for yield strength is based on the circular plate bending hypothesis, which converts the estimation problem from getting yield stress of the specimen to getting the plastic limit load of a circular plate under uniform pressure. The equation for ultimate tensile strength is based on a spherical cap assumption and plastic instability theory. 

### 3.1. Method for Yield Strength

**Figure 5:**
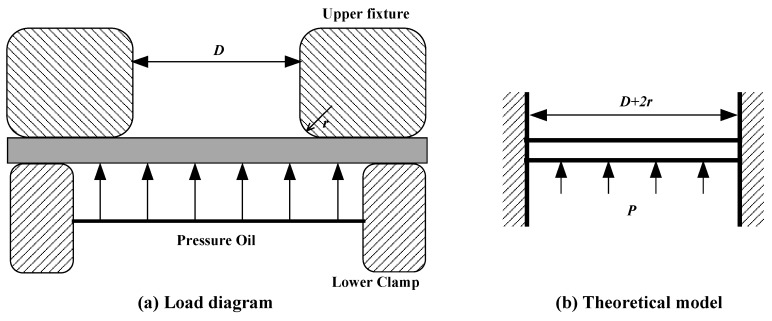
The mechanical model for yield strength. (**a**) Load diagram; (**b**) Theoretical model.

At the beginning of test, the pressure is relatively low, so the deformation of specimen is small, as shown in Figure 5a, and the model could be simplified as bending an edge-clamped circular plate under the action of normal pressure as shown in Figure 5b. The diameter of the circular plate is *D* + 2*r*, where *D* is the diameter of the upper fixture hole and *r* is the radius of the upper fixture chamfer. 

With increasing pressure, the bending moment becomes larger and larger. When the pressure reaches the plastic limit load of the structure, the plastic hinge is formed around the fixed edge, so the displacement will increase rapidly. Referring the plastic limit load equation of a fixed edge circular plate, we could get the equation for the structure shown in Figure 5b, which is written as

(1) $$P_{\text{lim}}=\alpha \sigma_{Y}{\left(\frac{t}{D+2r}\right)}^{2}$$

where $P_{\text{lim}}$ is the plastic limit load, *t* is the thickness of testing specimen [11], *D* and *r* are the parameters to describe the upper fixture, $\sigma_{Y}$ is the yield strength of the specimen and α is a constant parameter, which is only related to the material yield criterion, α=11.26 for Tresca yield criterion and α=12.17 for Von-Mises yield criterion [12]. By rewriting the Equation (1), we have Equation (2) for yield strength as

(2) $$\sigma_{Y}=\frac{1}{\alpha }{\left(\frac{D+2r}{t}\right)}^{2}P_{\text{lim}}$$

By Equation (2), we can get the yield strength of the testing specimen from the plastic limit load $P_{\text{lim}}$. Also, the upper fixture diameter *D* and the radius *r* shall be provided, which means that this testing method is fixture related. The optical microscope is used to measure the parameters *D* and *r* for each upper fixture, and α=12.17 for most steel used for pressure vessel. The plastic limit load $P_{\text{lim}}$ is calculated from the pressure-displacement curve as shown in Figure 4. The two times slope method is used in this paper to get the structure plastic limit load from the pressure-displacement curve. As illustrated in Figure 4, when the pressure is low, the displacement of the specimen is small and roughly proportional to the pressure, and when the load pressure reach a certain value, probably around 20 MPa, the displacement increases rapidly and is no longer proportional to the pressure. All the pressure-displacement curves for different materials show the same pattern, but the specific pressure value is different. Figure 4 shows that the pressure value of Q235 specimens is lower than those of Q345R or S30408.

### 3.2. Method for Ultimate Tensile Strength

With the increase of pressure, the plastic zones appear around the fixed boundary and expand. After the first plastic hinge, the plastic zone enlarges with the pressure increase. The deformation of the specimen is controlled by a bending moment which gives way to membrane stress, and the sphere cap assumption is used to describe deformation. 

**Figure 6:**
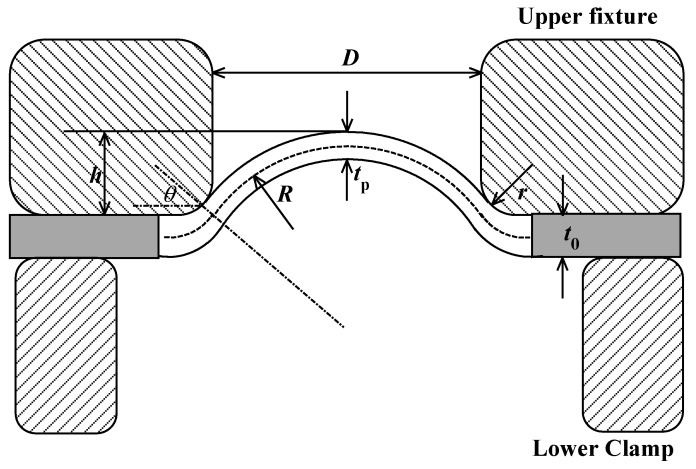
The mechanical model for ultimate tensile strength.

We assume that the cap is perfectly spherical in a small area at the top of the specimen with radius *R* and thickness $t_{P}$ According to the traditional hydraulic bulge test, the true stress and strain at the top of the sphere cap could be described by Equation (3) [13]

(3) $$\left\{ \begin{array}{l} \sigma =\frac{PR}{2t_{p}}\\ \varepsilon =\text{ln}(\frac{t_{0}}{t_{p}}) \end{array}\right.$$

where *P* is pressure, *R* is radius and $t_{p}$ is thickness of sphere cap as shown in Figure 6. The outer of the specimen is always tangent with the upper fixture, so we have equations

(4) $$\left\{ \begin{array}{l} r\;\text{cos}\;\theta +(R+\frac{t_{p}}{2})\text{cos}\;\theta =r+\frac{D}{2}\\ r(1-\text{sin}\;\theta )+(R+\frac{t_{p}}{2})(1-\text{sin}\;\theta )=h \end{array}\right.$$

Let *z* = *D*/2, and solve the Equation (4), we have the solution about *R*.

(5) $$\left\{ \begin{array}{l} R+\frac{t_{p}}{2}=\frac{h}{2}-r+\frac{{\left(r+z\right)}^{2}}{2h}\\ \theta =2\;\text{arctan}\left(\frac{z+r-h}{z+r+h}\right) \end{array}\right.$$

The volume of the specimen is a constant. The original volume of the specimen is $V_{0}=\pi {\left(z+r\right)}^{2}t_{0}$, for which *t*_0_ is the original thickness. There are two volume segments in the deformed specimen. One segment is the sphere cap and the other segment contacts with the upper fixture. For the sphere cap part, we assume that it has uniform thickness that is equal to $t_{p}$, so the volume of the sphere cap equals $V_{sp}=2\pi R^{2}(1-\text{sin}\;\theta )t_{p}$. For the contact part, we assume that there is a linear relation between the thickness and the contact angle θ, then, thickness of the contact-part could be described as

(6) $$t(\varphi )=t_{p}+\frac{\varphi -\theta }{\frac{\pi }{2}-\theta }\left(t_{0}-t_{p}\right)$$

where φ is contact angle, θ≤φ≤π/2 when $\begin{array}{cc} \varphi =\pi /2, & t=t_{0} \end{array}$ and $\begin{array}{cc} \varphi =\theta , & t=t_{p} \end{array}$. By using integration method to calculate the contact segment volume, we can get the thickness after deformation

(7) $$t_{p}=\frac{p-\pi {\left(r+z\right)}^{2}}{2\pi \left(\text{sin}\left(2\;\text{arctan}\left(\frac{r-h+z}{r+h+z}\right)\right)-1\right){\left(\frac{h}{2}-r+\frac{{\left(r+z\right)}^{2}}{2h}\right)}^{2}-p}t_{0}$$

where

$$p=\pi r(r+z)\left(\frac{\pi }{2}-2\;\text{arctan}\left(\frac{r-h+z}{r+h+z}\right)\right)+\pi r^{2}\left(\text{sin}\left(2\;\text{arctan}\frac{r-h+z}{r+h+z}\right)-1\right)$$

For simplification, Equation (7) can be written as

(8) $$t_{p}=f(h,z,r)t_{0}$$

There are three parameters in the function *f*(*h*, *z*, *r*). *r* and *z* are related to the dimensions of upper fixture and *h* is the deformation of the specimen. By applying Equations (5) and (7) to Equation (3), we obtain the approximate strain-stress relationship of the small specimen. 

During the conventional tensile test, necking starts at the maximum load, which means δF=0. At the start of necking, the plastic instability occurs, and this criteria can also be used in the small specimen hydraulic bulge test. For the conventional tensile test, the plastic instability occurs when the true stress and true strain have the relation

(9) $$\sigma_{T}=\frac{\delta \sigma_{T}}{\delta \varepsilon_{T}}$$

The plastic instability principle from the conventional tensile test as shown in Equation (9) is used for HBT. Combining Equation (8) and the stress and strain Equation (3) for the sphere cap into Equation (9), we have the plastic instability criteria for the hydraulic bulge test. The plastic instability will occur when

(10) $$\frac{dP_{c}}{dh_{c}}=KP_{c}$$

where *P_c_* is the critical pressure and *h_c_* is the critical height, and we have

(11) $$K=\frac{1}{h}-\frac{2(h-z)}{h^{2}+r^{2}+z^{2}-2r(h-z)}$$

Thus, the ultimate tensile strength of the specimen is

(12) $$R_{m}=\frac{P_{c}R_{c}}{2t_{p}}\left(1+\text{ln}\left(\frac{t_{0}}{t_{p}}\right)\right)$$

Equation (10) is used to determine the plastic instability point in the pressure-displacement curve, and the data of the point is used to calculate the ultimate tensile strength by Equation (12). One thing that must be noted is that the calculated strain and stress are the true strain and true stress, and for the conventional tensile test, the ultimate tensile strength is engineering stress. Therefore, the true stress has to be converted to engineering stress, if needed.

By comparing Equation (2) for the yield strength and Equation (11) for ultimate tensile strength, Equation (2) is related to the second order of the original thickness while Equation (12) is related to the first order of the thickness. Thus, the pressure displacement curve cannot be normalized to strain-stress curve as the conventional tensile test. This is because that deformation mechanism has changed from plate bending at the beginning to membrane extension at the last moment. The low pressure part of the pressure-displacement curve is used for yield strength estimation and the high pressure part of the curve is used for ultimate tensile strength. 

**Figure 7:**
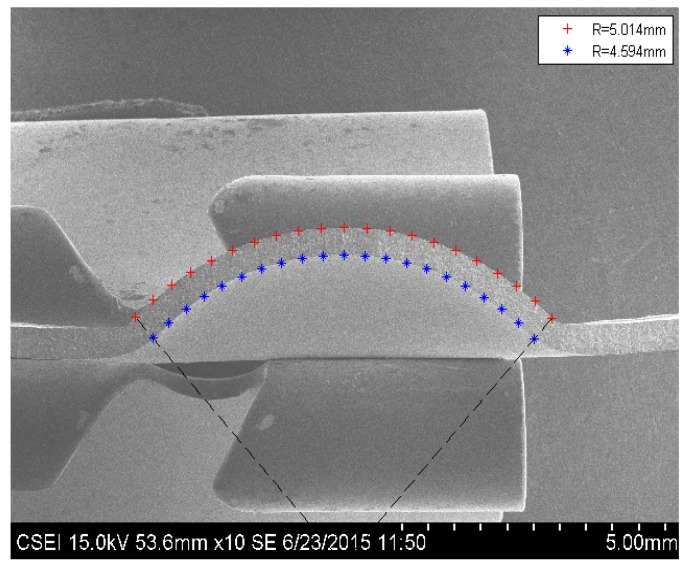
The stainless-steel specimen section at 100 MPa.

In order to prove the sphere cap with uniform thickness assumption, a stainless-steel specimen with thickness 0.48 mm is loaded to 100 MPa to make a permanent deformation and then unload. Subsequently, the specimen was cut into two pieces. One of the sections is shown in Figure 7. Clearly, the shape of the section is sphere cap, with inner radius 4.549 mm and outer radius 5.014 mm. The thickness of the sphere crown is 0.42 mm, 12.5% thinner than the original specimen. The sphere cap has a much larger surface area than the circular plate, so the thickness is reduced from 0.48 to 0.42 mm. Figure 7 illustrates that the sphere cap with the uniform thickness assumption is reasonable.

## 4. Comparison with Conventional Tensile Testing Methods

To verify the HBT testing results, two different types of materials, a low carbon steel Q345R and a stainless steel S30408, are chosen. Each type of material is tested by both conventional tensile test and hydraulic bugle test. The test results from the former method are used to verify the HBT results, and the results is listed in Table 1. For Q345R, there are four specimens used for the conventional tensile tests, and 36 specimens for the hydraulic bulge test, for which the thickness is from 0.490 to 0.510 mm. For S30408, there are two specimens used for the conventional tensile tests, and 25 specimens for the hydraulic bulge test with a thickness of 0.48 mm. 

**Figure 8:**
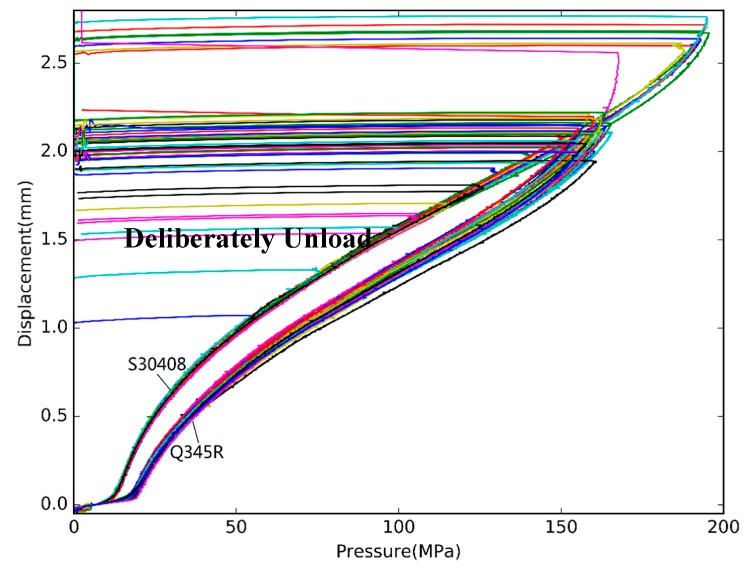
The pressure-displacement curve of Q345R and S30408.

**Figure 9:**
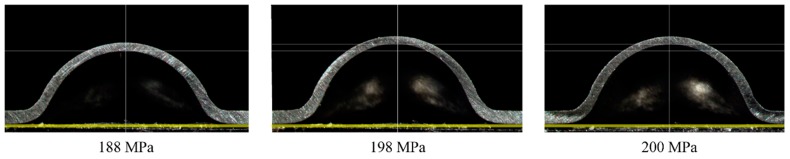
The cross sections of the specimens (S30408, 188, 198 and 200 MPa).

All the pressure-displacement curve is shown in Figure 8. It can be seen that almost all the curves of S30408 coincide when the pressure is lower than 55 MPa. In order to check the cross-sections of specimens, as shown in Figure 9, some HBTs are intentionally unloading to zero after the pressure was larger than 55 MPa. Although the trend and pattern are similar, the curves of Q345R are still significantly different to S30408. 

**Figure 10:**
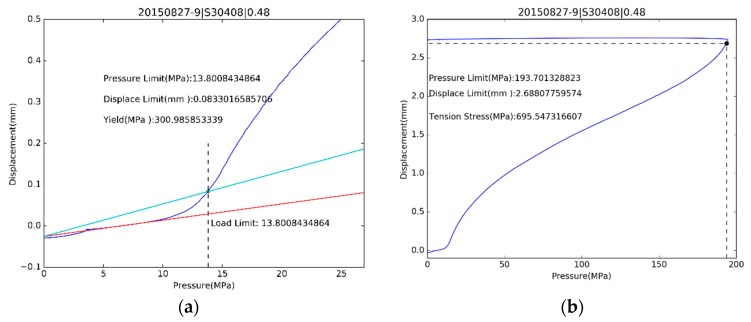
The estimation for (**a**) yield stress and (**b**) ultimate tensile strength of S30408.

**Figure 11:**
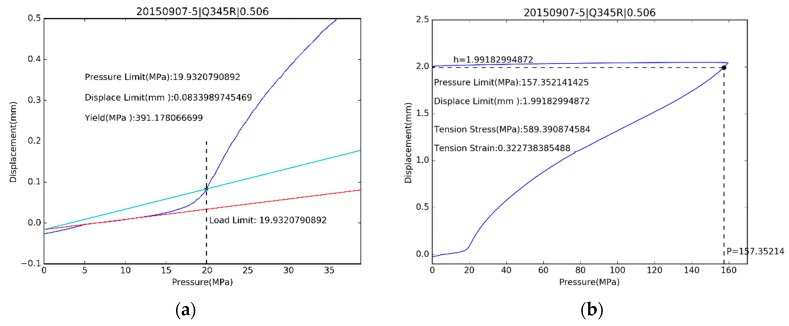
The estimation for (**a**) yield stress and (**b**) ultimate tensile strength of Q345R.

One example of estimated yield strength for S30408 is shown on the left side of Figure 10, and Q345R in Figure 11. Ultimate strength estimations are shown on the right side of Figure 10 for S30408 and Figure 11 for Q345R.

**Figure 12:**
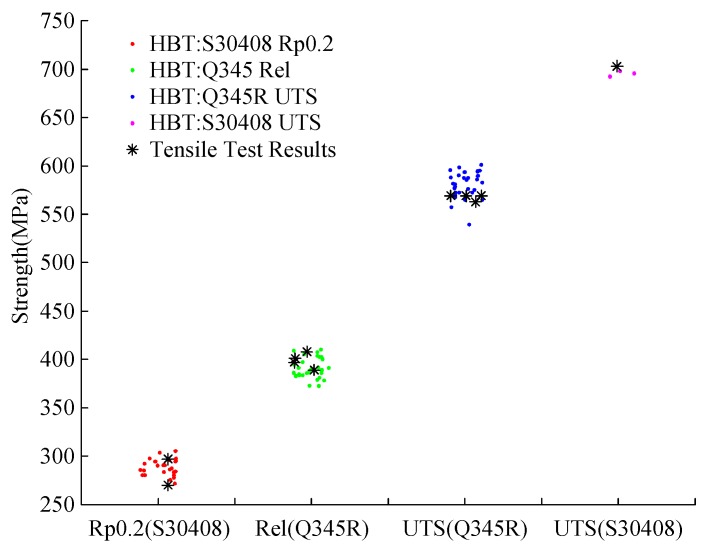
Comparing the results from HBT with conventional tensile test.

Both the testing results from conventional tensile test and hydraulic bugle test are shown in Figure 12. By comparison of test results, the HBT method and the estimation equations are reliable. All parameters used in the estimation have physical meaning.

**Figure 13:**
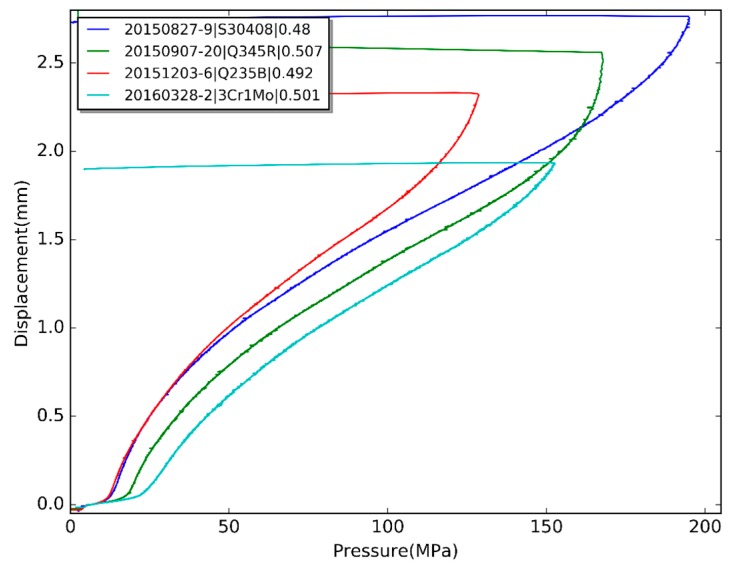
The HBT curves of four different materials.

In order to illustrate the strength estimation procedure, four different materials are tested by HBT—Q235B, Q345R, S30408 and 3Cr1Mo. The pressure-displacement curves are shown in Figure 13, and the data are listed in Table 2.

## 5. Conclusions

A small specimen testing method, called hydraulic bulge test (HBT), is proposed with theoretical derivations for yield strength and ultimate tensile strength. This test method is reliable. Both thickness of specimen and size of fixture are considered in the theoretical equations. The estimation procedure to calculate material properties do not need to carry out a large number of experiments or time-consuming finite element simulations. Theoretical models are used to establish the strength estimation procedure, and the parameters are the thickness of the specimen and the geometry of the fixture, which can be directly measured, and there are no empirical expressions. The theoretical equations may help us to come to a better understanding about the small hydraulic bugle test and lead us to a better design of the testing equipment. The hydraulic bulge test is one of the most prospective methods among the small specimen testing techniques. 

The defects of the HBT are the temperature range and testing device. The temperature range for hydraulic bulge test is small, and the hydraulic testing system is more complex than small punch test, which contains pump, piping system, sensors, fixtures, etc.

## Figures and Tables

**Table 1:** The conventional tensile test results of Q345R and S30408.

Material	Units	Q345R				S30408
Specimen No.		1	2	3	4	1
Yield Strength (YS)	MPa	408	389	401	397	297
Ultimate Tensile Strength (UTS)	MPa	569	563	569	569	703

**Table 2:** The data for strength estimation.

Specimen		Yield Strength		Ultimate Tensile Strength		
Material	Thickness (mm)	*P*_lim_ (MPa)	YS (MPa)	*P_c_* (MPa)	*h_c_* (mm)	UTS (MPa)
S30408	0.480	13.8	301	194	2.69	695
Q345R	0.506	19.3	391	157	1.99	589
Q235B	0.492	12.6	262	123	2.08	461
3Cr1Mo	0.501	25.8	517	136	1.63	580
